# Direct Samples of Interstellar and Interplanetary Material with IMAP

**DOI:** 10.1007/s11214-026-01291-w

**Published:** 2026-04-02

**Authors:** J. R. Szalay, E. Provornikova, E. Ayari, M. Bzowski, E. R. Christian, H. O. Funsten, A. Galli, M. Gkioulidou, M. Horányi, S. Livi, D. J. McComas, E. Möbius, K. Ogasawara, F. Rahmanifard, J. S. Rankin, D. B. Reisenfeld, N. A. Schwadron, Z. Sternovsky, P. Swaczyna, D. Turner, G. P. Zank, E. J. Zirnstein

**Affiliations:** 1 Department of Astrophysical Sciences, Princeton University, Princeton, NJ 08540 USA; 2 The Johns Hopkins University Applied Physics Laboratory, Laurel, MD 10587 USA; 3 University of Colorado Boulder, Boulder, CO 80303 USA; 4 Space Research Centre PAS (CBK PAN), 00-716 Warsaw, Poland; 5 Heliophysics Science Division, NASA Goddard Space Flight Center, Greenbelt, MD 20771 USA; 6 Space Research and Planetary Sciences, Physics Institute, University of Bern, Bern, Switzerland; 7 Southwest Research Institute, San Antonio, TX 78228 USA; 8 Space Science Center, University of New Hampshire, Durham, NH 03824 USA; 9 Los Alamos National Laboratory, Los Alamos, NM 87545 USA; 10 Department of Space Science, University of Alabama in Huntsville, Huntsville, AL 35899 USA

**Keywords:** Interstellar medium, Interstellar dust, Interstellar neutral atoms, IMAP

## Abstract

The Interstellar Mapping and Acceleration (IMAP) mission probes the interaction between our heliosphere and the interstellar medium (ISM) in unprecedented detail. The broad science that IMAP addresses has been organized into three distinct science themes: A) Acceleration and the broader context of the solar wind and space weather; B) Exploring the outer heliosphere through energetic neutral atoms, and C) Sampling of the interstellar and interplanetary material. This paper summarizes the scientific goals of the latter theme and identifies the key scientific opportunities for IMAP available due to its unique ability to directly sample the interstellar and interplanetary material from 1 au. It is organized into three broad scientific questions that directly relate to IMAP’s science objectives: 1) What is the state of the pristine upstream LISM and how does it relate to its origins and evolution? 2) How does the VLISM interact with the heliosphere? 3) How does interstellar material, as well as interplanetary dust, affect the near-Sun environment?

## Introduction

The Interstellar Mapping and Acceleration Probe (IMAP) mission (McComas et al. 2025) probes the interaction between our heliosphere and the interstellar medium (ISM) in unprecedented detail. With a suite of 10 scientific instruments, it creates comprehensive maps of the Sun’s interaction with the surrounding interstellar material, including measurements of the solar wind plasma, suprathermal, and high-energy particles, interplanetary magnetic fields, backscattered solar Lyman-$\alpha $ radiation, interstellar neutrals, and dust grains from both interstellar and interplanetary space. The mission has four primary Science Objectives: *O1) Improve understanding of the composition and properties of the local interstellar medium; O2) Advance understanding of the temporal and spatial evolution of the boundary region in which the solar wind and the interstellar medium interact; O3) Identify and advance understanding of processes related to the interactions of the magnetic field of the Sun and the local interstellar medium; O4) Identify and advance understanding of particle injection and acceleration processes near the Sun, in the heliosphere and heliosheath.*

**Fig. 1:**
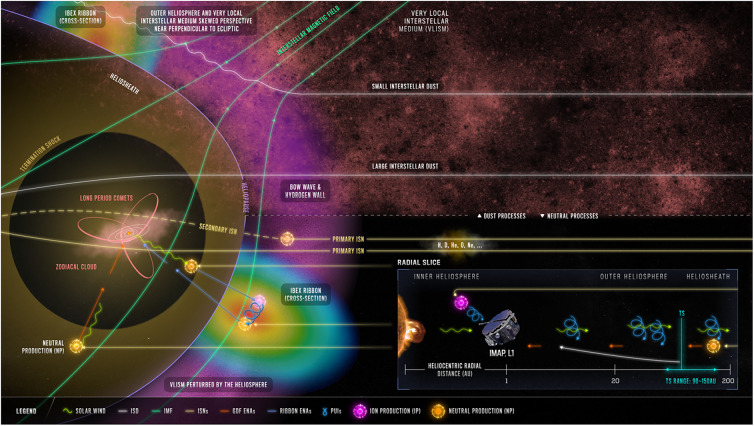
Overview of the interaction between the local interstellar medium (LISM) and our heliosphere. The pristine LISM plasma flows from right to left, where it forms a bow wave, a hydrogen wall, and is deflected around the heliopause. Interstellar neutrals can enter the heliosphere and be detected by IMAP. A fraction of these interstellar neutrals charge exchange with solar wind ions, which produces anti-sunward moving neutral solar wind particles that can subsequently charge exchange again in the heliosheath, or outside the heliopause, to be detected by IMAP to probe our heliosphere’s boundary, including the IBEX Ribbon. Interstellar neutrals and plasma are kinematically decoupled outside the heliopause and interact by charge exchange, producing a secondary component of interstellar species, inflowing inside the heliopause and slightly deviated from the unperturbed flow direction. Interstellar dust grains also flow in a direction similar to that of the interstellar neutrals, and their ability to penetrate or be deflected by the heliosphere depends on their size and charge. Small nanometer-sized grains are primarily deflected, while larger submicron- to micron-sized grains can transit the heliosphere and be detected by IMAP. Adapted from the summary figure in McComas et al. (2025)

Here, we focus primarily on IMAP objectives O1 and O3, and summarize the key scientific opportunities for IMAP by directly sampling the interstellar and interplanetary material from 1 au. Figure 1 summarizes the various phenomena discussed in this study. We identify three broad scientific questions in each Section that directly relate to IMAP’s science objectives: Sect. 2) What is the state of the pristine^1^ upstream LISM, and how does it relate to its origins and evolution? Sect. 3) How does the VLISM interact with the heliosphere? Sect. 4) How does interstellar material, as well as interplanetary dust, affect the near-Sun environment? Subsections provide more focused scientific questions, with tables that include relevant questions, outline which IMAP measurements would help advance these questions, and discuss modeling inputs that would help reach closure on the science questions.

## What Is the State of the Pristine Upstream LISM and How Does It Relate to Its Origins and Evolution?

The composition of the pristine LISM provides key information about the evolution of matter in our galaxy from the Big Bang to today. Big Bang Nucleosynthesis set the elemental and isotopic composition of all matter in the universe at its beginning, with only H and He isotopes and some traces of Li as initial conditions (Copi et al. 1995; Coc et al. 2004), whose abundances can be tied to the baryon density of the universe. Solar system abundances froze in the state of matter locally in our galaxy at the birth of the Sun about 4.6 billion years ago as compiled from photospheric observations (Asplund et al. 2005) and meteoritic samples (Lodders 2003). The LISM with its neutral gas and dust features the composition of galactic matter in our immediate neighborhood at the present time.

These three points in time place constraints on the evolution of matter in the universe and specifically within our galaxy. While there has been significant progress in constraining the abundances in the early universe (Turner 2022), allowing for a detailed account of solar system abundances, observational evidence of the abundances in our interstellar galactic neighborhood remains challenging, especially for heavy elements and their isotopes.

Another essential aspect of the LISM just outside the heliosphere is its dynamical state or its flow vector, which, along with the solar wind from inside and the interstellar magnetic field, controls the size and shape of the heliosphere. In comparison with the dynamics of the surrounding interstellar clouds based on absorption line observations (Redfield and Linsky 2008; Linsky and Redfield 2014) the realization has emerged that the solar system may be within the interaction region of two local interstellar clouds (Swaczyna et al. 2022b). Finally, the kinetic state of the LISM is of fundamental interest, which can be obtained from the velocity distributions of detectable interstellar neutral (ISN) species. The possibility of cloud interaction right at our door step has made this topic even more compelling.

IMAP provides a well-equipped platform to monitor material from the LISM transiting the heliosphere as it will measure interstellar neutral atoms, pickup ions (PUIs), and dust grains. In this section, we outline how IMAP measurements can improve our understanding of the upstream, pristine LISM. These observations will allow for a better understanding of the initial formation conditions of our solar system and those in the local Galactic matter that the Sun is currently transiting, and how the interstellar medium interacts with our heliosphere.

### What Is the Elemental and Isotopic Composition of the ISM?

The isotopic abundances of the light ions, namely D/H and ^3^He/^4^He provide a critical window into the formation of matter in the Big Bang Nucleosynthesis. Specifically, constraints on these ratios provide insight in a complementary way to existing measurements on the physical state during the critical first few minutes after the Big Bang (Geiss and Reeves 1972; Schramm 1998; Copi et al. 1995). Separately, heavy elemental abundances of O and Ne isotopes can inform us about the processing of matter in stars at later stages in time after the Big Bang (Prantzos 1998; Geiss et al. 2002).

**Fig. 2:**
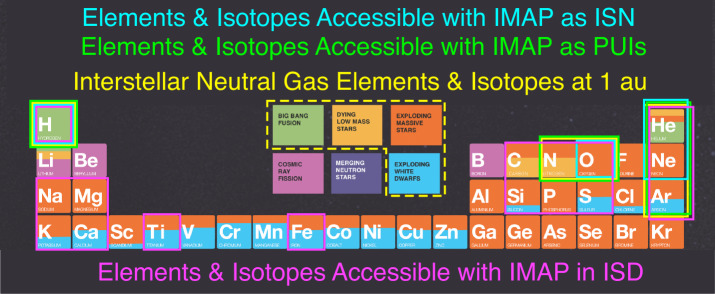
First four rows of the periodic table of elements color-coded according to astrophysical sources of these elements in the universe. Those elements and their respective isotopes that the ISN flow carries to 1 au are framed in yellow. The yellow frame with a dashed line indicates the astrophysical processes accessible through the ISN. The turquoise frames indicate the elements that IMAP-Lo can see directly as ISNs. The green frames indicate the elements SWAPI and CoDICE can see through PUIs. Some of these elements and isotopes in the ISN are also present in interstellar dust (ISD). In addition, many refractory elements ionized in the ISM and thus not accessible directly at 1 au are embedded in the ISD. Hence, ISD observations by IDEX enable constraints on the elements and isotopes framed in magenta. (Adapted from Chemistry & the Universe, https://chandra.harvard.edu/resources/illustrations/chemistry_universe.html)

Figure 2 shows a schematic representation of the lower half of the periodic table of elements, a fraction of which can be measured as neutral interstellar gas or interstellar dust grains. The elemental boxes are color-coded according to their fractional origin from different production processes. Green denotes production during Big Bang nucleosynthesis, which applies to H, He, and some fraction of Li and their isotopes (Copi et al. 1995; Coc et al. 2004). Yellow indicates an origin from dying low mass stars, deep orange from supernova type II explosions, and cyan from supernova type Ia explosions (Thielemann et al. 2018). Magenta indicates production through cosmic ray spallation processes and purple through neutron star mergers, which are both of no significance here. Be and B, are studied through galactic cosmic rays, and the latter process is only relevant to very heavy elements beyond Kr, which are currently not accessible to in situ studies of the interstellar medium. However, it is important to note that elements with contributions from four of the production processes (all except cosmic ray spallation and neutron star mergers), arrive at 1 AU as neutral interstellar gas (yellow border in Fig. 2) and thus can be measured directly as neutral atoms with IMAP-Lo (Schwadron et al. 2025) and through PUIs with CoDICE (Livi et al. 2026) and SWAPI (Rankin et al. 2025).

H and He derive mostly from the Big Bang, O and Ne mostly from SN Type II, but N partially from SN Type II and low mass stars and Ar partly from SN Type II and SN Type Ia. In addition to H, N, and O, interstellar dust grains provide access to refractory elements, such as C, Na, Mg, Si, P, S, K, Ca, Ti, and Fe, which show increasing contributions from SN Type Ia with mass. Any similarities and differences between the abundances of these elements in the solar system and the Very Local Interstellar Medium (VLISM) provides important new constraints on the evolution of matter in our galaxy through the accumulation of material from the different stellar death scenarios and thus on models of matter evolution in the galaxy.

Thus far, knowledge of light element isotopic abundances of the ISM has primarily been based on remote observations. The D/H ratio in the ISM has been obtained mostly through absorption spectroscopy using the lines of nearby stars. This technique is subject to systematic uncertainties due to measuring the line-of-sight integral values of the absorption (Linsky et al. 2006) and various potential depletion processes in the interstellar medium, including interstellar dust. According to Linsky et al. (2006), “the most direct test of the deuterium-depletion model would be to measure (D/H) dust in interstellar dust grains.” Keller et al. (2000) performed such an experiment by measuring the deuterium abundance in interplanetary dust particles containing amorphous carbonaceous material, where the relation to interstellar matter is inferred. A first attempt to obtain D/H in-situ from interstellar neutral gas was made with IBEX (McComas et al. 2009b) data (Rodríguez Moreno et al. 2013), albeit with very large statistical and systematic uncertainties. A discussion of the detectability of ISN D by IMAP-Lo, provided by Kubiak et al. (2024), suggests that the collected statistics of ISN D atoms will be significantly larger owing to longer collection time during the year due to different observation geometry, provided by the IMAP-Lo ability to adjust the elongation angle of its boresight relative to the spin axis.

Information on the ^3^He/^4^He ratio in the ISM is available from spectroscopy of H II regions (Bania et al. 2002). Values for the protosolar nebula have been derived from observations in the solar wind (Bodmer and Bochsler 1998) and in the Jovian atmosphere (Mahaffy et al. 1998) for comparison along the evolution timeline. The first direct observation of ^3^He/^4^He in the VLISM stems from PUI measurements by SWICS on Ulysses (Gloeckler and Geiss 1998b), with somewhat refined values after that (Gloeckler and Geiss 1998b). These estimates for the ^3^He/^4^He ratio were higher than in the protosolar nebula and the constraints in H II regions. However, the measurement of the ratio from interstellar gas captured on foils with the COLLISA experiment (Busemann et al. 2006) indicated a value close to those from PUI observations. Yet, the combined relatively large statistical and systematic uncertainties of Ulysses’ PUI measurements still allow the value to overlap with that from the foil experiment. Therefore, IMAP’s PUI measurements by CoDICE and SWAPI with substantially higher fidelity will be crucial for determining the ^3^He/^4^He ratio in the LISM.

Both the in situ observations of D/H and ^3^He/^4^He carry large uncertainties. IMAP can substantially reduce these uncertainties to provide tighter constraints on interstellar abundances. Since IMAP observes at 1 au, abundance changes due to differential ionization and, in the case of the H isotopes, differential radiation pressure must be taken into account. This issue of different transport and loss pathways for different isotopes is less important for He isotopes because their ionization rates are similar to each other, and radiation pressure is largely negligible for He (Kowalska-Leszczynska et al. 2023), however it is essential to constrain for H isotopes (Kowalska-Leszczynska et al. 2018a,b, 2020). Careful modeling of these effects, aided by better understanding of the interstellar H flow relative to He becomes essential, as currently there are still large uncertainties in understanding the H flow observations at 1 au (Schwadron et al. 2013; Katushkina et al. 2015b; Rahmanifard et al. 2019). IMAP provides extensive opportunities to observe ISN H during almost entire year, including opportunities to separate the primary and secondary populations (Kubiak et al. 2024), which will facilitate understanding the flow of ISN H inside the heliosphere and processes modifying its flow in the VLISM.

With respect to heavy elements, the local interstellar Ne/O abundance has been measured through PUIs (Gloeckler and Fisk 2007) and through direct ISN observations (Park et al. 2014), with results that agree with each other, but indicate a value approximately a factor of 2 higher than the abundance ratio in the solar system, as obtained from solar energetic particles (Reames 1998), in the solar wind (Bochsler 2007), and for EUV lines in the photosphere (Young 2005). These solar values appear to be substantially lower than those consistent with a helioseismological determination, which also agrees with EUV measurements in 21 stellar atmospheres (Drake and Testa 2005). On the other hand, a comparison with interstellar X-ray observations indicates a lower ratio more consistent with observations in the solar atmosphere (Juett et al. 2006). Overall, there appear to be several conflicting and puzzling results, which call for substantially improved measurements of the VLISM heavy elemental abundances.

IMAP is posed to reduce the large uncertainties in measurements of heavy element abundances. The reason for the apparent depletion of O over Ne is unknown. Potential reasons could be: (i) more O is locked in dust grains than Ne; (ii) there could be a general depletion pattern due to the motion of the Sun from its birthplace. IDEX observations of interstellar dust composition allow us to explore the heavy element abundances locked in ISD. Table 1 summarizes the measurements from different instruments on IMAP that will be crucial to advance our understanding of the VLISM composition and element abundances.

**Table 1:** Relevant science questions, IMAP measurements, and model inputs for Sect. 2.1

Relevant questions	What is the D/H abundance in the LISM? How does the directly sampled D/H abundance compare to that previously derived from remote observations? What are the isotopic ratios ^3^He/^4^He and ^22^Ne/^20^Ne in the ISM flow? What is the Ne/O abundance in the interstellar neutral atoms and dust grains? Why does the ISD mass range from Ulysses differ from expectations (Fig. 3)? Why do the Stardust and Cassini ISD composition measurements differ?
IMAP Measurements	IMAP-Lo: Direct sampling of H, D, O, Ne, and N atoms. D/H, Ne/O, ratio,. CoDICE: isotopic ratio of pick-up ions to infer neutral abundances: He, N, O, Ne, ^3^He/^4^He ratio, ^22^Ne/^20^Ne, Ne/O. IDEX: Mass distribution of ISD. Isotopic ratios, including Mg/Fe abundance in ISD.
Model input	Elemental and isotopic ratios as a function of different ISM evolution conditions. The effects of evaporation and sputtering from dust as well as the extent to which the solar wind can modify grain composition. Size- and composition-dependent filtration of ISD to compare the 1 au observations to the pristine ISM.

The different composition measurements taken with IMAP are highly complementary to each other. IMAP-Lo directly samples the ISN species while IDEX samples ISDs. IMAP-Lo offers opportunities to observe ISN O and Ne for more than 6 months each year, and to observe ISN Ne with the contribution to the signal from ISN O much reduced (Kubiak et al. 2023). CoDICE obtains the PUI distributions of He through Ne, which, depending on species, have different contributions from ISNs and inner source dust distributions (discussed in Sect. 4). The inner source of PUIs has been attributed to a variety of dust-related phenomena and is discussed in more detail in Sects. 4.1, 4.2. When reporting LISM abundances based on PUIs, any contribution from the inner source must be subtracted first. On the other hand, inner source PUIs provide valuable information about interplanetary dust particles and their interaction with the solar wind.

Fortunately, there is a clear path to discriminate between the two contributions to the PUI distributions. While the LISM neutrals are prevalent in the part of the PUI velocity distribution close to the cut-off, the inner source is more prominent in the distribution closer to the core solar wind. Because C has a low first ionization potential and thus is completely ionized in the ISM, it is not found in the ISN flow through the solar system, and its presence in PUI distributions provides a clean sample of inner source PUIs for a quantitative comparison. IMAP can provide further constraints to help disentangle the multiple potential origins of the inner source of PUIs (e.g. Schwadron et al. 2000; Wimmer Schweingruber and Bochsler 2003), as discussed further in Sect. 4.2. The parent material of this inner source most likely stems from interplanetary and interstellar dust, which provides another very important source of information about the ISM and solar system material and will be discussed next.

### How Does the ISM Compare with Interstellar and Interplanetary Dust?

Interstellar dust (ISD) plays a critical role in the formation of stars and their planets, and in the chemistry and dynamics of interstellar matter (Draine 2009). These grains are believed to be the primary carriers of condensable interstellar matter, with an elemental composition that complements that of the interstellar gas, as a significant fraction of heavy elements in the ISM is sequestered within interstellar dust grains. ISD particles’ surfaces enable catalytic chemical reactions, emit and absorb radiation, act as a source and sink of electrons and ions, and couple the interstellar gas to magnetic and radiation forces.

**Fig. 3:**
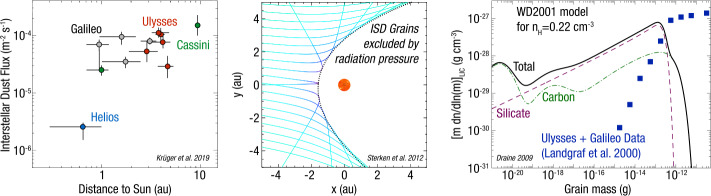
Interstellar dust (ISD) in the inner heliosphere. a) ISD flux as a function of heliocentric distance, as detected by multiple missions to date (Krüger et al. 2019). b) Representative trajectories illustrating how the solar radiation pressure can exclude ISDs from the inner heliosphere (Sterken et al. 2012). c) Discrepancy in the distribution of ISD mass between a model incorporating silicate and carbonaceous grains that is consistent with remote-based observations in black (Draine 2009) and in-situ-based observations in blue (Landgraf 2000)

The flow of ISD through our solar system was first identified with a detector onboard the Ulysses spacecraft during the first years of the mission (Grün et al. 1992). These in situ measurements created a still-unresolved conflict between the mass distributions inferred from Ulysses with those from decades of optical observations of the attenuation and polarization of the light from nearby stars (Draine 2009; Wang et al. 2015). The many large grains detected in situ by Ulysses require a mass in the condensable elements that is much higher than what would be compatible with stellar extinction data. This conflict is illustrated in Fig. 3, which shows the flux of interstellar dust and its interaction with the heliosphere, where a fraction of this material makes its way to 1 au where IMAP measures it.

**Fig. 4:**
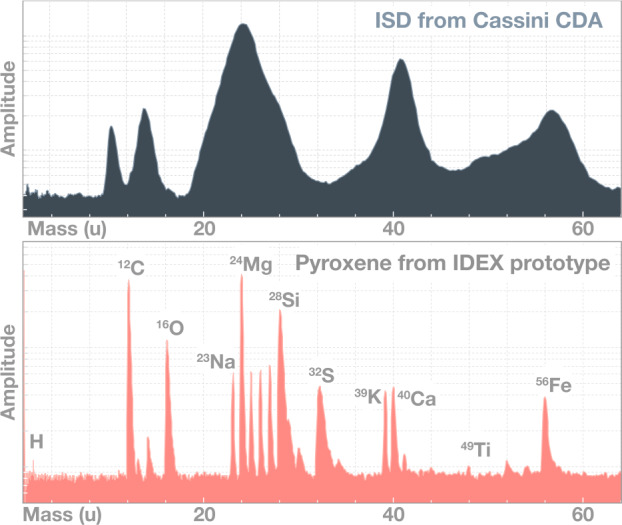
Impact ionization mass spectrum of an ISD particle recorded by Cassini CDA (top) (Altobelli et al. 2016). Mass spectrum recorded by IDEX prototype instrument in the laboratory (bottom) of a Pyroxene mineral particle ((NaCa)(Mg, Fe, Al)(Al, Si)_2_O_6_ – Sodium Calcium Magnesium Iron Aluminum Silicate). The carbon peak is due to contamination from sample preparation

To date, two missions have measured the composition of ISD in the solar system, yielding contradictory results. The Stardust mission collected seven particles which were identified as ISD grains, returning them to Earth for laboratory study (Westphal et al. 2014). The Cassini mission measured the compositions of three dozen interstellar particles while orbiting Saturn (Altobelli et al. 2016). The Stardust ISD samples exhibit a wide range of elemental compositions, crystalline structures, and sizes (Westphal et al. 2014), while all Cassini ISD samples consist of Mg-rich silicates, exhibiting Mg/Fe and Mg/Ca ratios within narrow bands. To resolve this discrepancy and improve understanding of ISD composition and properties, the IDEX instrument will measure the mass, composition, and flux of a significantly larger particle sample with an enhanced capability to distinguish different elements and isotopes within the grains. Figure 4 compares the mass resolution of Cassini CDA and IDEX. IDEX results were obtained using a dust accelerator (Horányi et al. 2025).

Interplanetary dust particles (IDPs) originate primarily from material shed by short-period or Jupiter-family comets, whose orbits are influenced near aphelion by the gravity of our solar system’s largest planet. The fraction of IDP originating from asteroids is not yet well understood. However, approximately 10% of the IDP flux is believed to be of asteroidal origin (Nesvorný et al. 2011), and an even more uncertain and likely smaller fraction comes from long-period comets (Nesvorný et al. 2010). IDEX measurements of IDPs sample material originating from a large number of comets. By analyzing the composition of ISD and IDPs, we can answer the question of how the material from the interstellar medium compares with that of comets. Such constraints can further inform on our understanding of the formation of our solar system (e.g. Cleeves et al. 2014).

IDEX measures elemental abundances, for example Si, Mg, Fe, C, H, O, N, S, Al, Ca, Na and Ni, which together make up 99.2% of the mass of chondritic meteorites (McSween et al. 2022; Lodders 2003). Elemental, isotopic, and molecular compositions reveal: (a) the makeup of interplanetary dust and interstellar dust from the local interstellar cloud; (b) the extent to which cometary material was processed in the solar nebula; and (c) the origins of the solar system’s refractory organic matter. Direct measurements comparing interplanetary and interstellar dust grain compositions will allow us to determine whether the contemporary local interstellar cloud dust population has properties consistent with the feedstock that formed the solar system. These populations will be distinguished by the annual modulation of ISD detections throughout the year and via the speed-dependent compositional features that enable impact speed discrimination between the two populations (Horányi et al. 2025). IDEX can also determine whether the JFC fine-grained components preserve unprocessed molecular cloud particles or show signatures of processing in the solar system. Finally, IDEX observations can help determine whether primitive bodies’ organics share a common source or formed from distinct reservoirs.

The elemental ratios within each grain also provide important clues. IDEX measures the elemental ratios H/C, O/C, N/C, and C/Si, and the possible presence of major organic functional groups such as benzene (C_6_H_6_), carboxyls (R-COOH), and amines (R-NH2). The detection of complex organics present in ISDs would give important clues on ISD grain evolution in the ISM (Bouwman et al. 2023). Additionally, the H/C ratio provides information on the degree of aromatic bonding, O/C informs on the availability of oxygen during the material’s formation, N/C provides clues on the temperature and the presence of water as the organics formed, and C/Si indicates the organic-to-silicate ratio. The combination of functional groups present reveals the degree of chemical complexity of the organic material (Alexander et al. 2017). Such molecular fragments also allow partial reconstruction of the original, potentially much larger, parent molecules, which also may have important bearing on the key ingredients for habitability (Postberg et al. 2018). IMAP can directly measure these compositional ratios and Table 2 summarizes the relevant questions, IMAP measurements, and model inputs that would address these topics.

**Table 2:** Relevant science questions, IMAP measurements, and model inputs for Sect. 2.2

Relevant questions	How does the current ISM composition compare to the material from which the solar system formed? Does the ISM composition match solar abundances (e.g. Draine 2009)? Are complex organics present in ISDs (e.g. Bouwman et al. 2023)?
IMAP Measurements	IDEX: Measures the mass, composition, and flux of interstellar dust particles. (Altobelli et al. 2016; Kempf et al. 2023) IMAP-Lo: Measures abundances of He, O, Ne, D relative to H in the interstellar medium surrounding the heliosphere today.
Model input	ISD filtration as a function of size and composition, both at the heliospheric boundary and throughout the heliosphere due to focusing/defocusing effects (e.g. Landgraf 2000). Modification of abundances interstellar neutrals inside the heliosphere due to re-ionization (Kubiak et al. 2023, 2024)

### Is ISD Destroyed and Created Throughout the ISM or Does It Carry the Imprint of Its Origins?

To explain the very low abundances of ISN heavy elements compared to solar abundances, ISD has been strongly implicated in sequestering much of the heavy elements within the ISM (Draine 2009). This sequestration relies on the continual destruction and condensation of dust grains within the ISM, which are suggested to be catastrophically destroyed by interstellar shocks and re-accreted multiple times in the ISM. Recently updated models of grain formation that account for extinction, emission, and polarization of ISD within the ISM find that a single composite “astrodust” grain containing silicate and carbonaceous material is most consistent with observations (Hensley and Draine 2023). This updated analysis based on decades of modeling and observations, can now be directly tested with IMAP, namely by determining if most grains have a mixture of carbonaceous and silicate material, or if there are primarily separate carbonaceous and silicate grain types. We note that the Cassini ISD observations did not find evidence for an appreciable amount of carbonaceous material in the ISD grains it had identified (Altobelli et al. 2016).

**Fig. 5:**
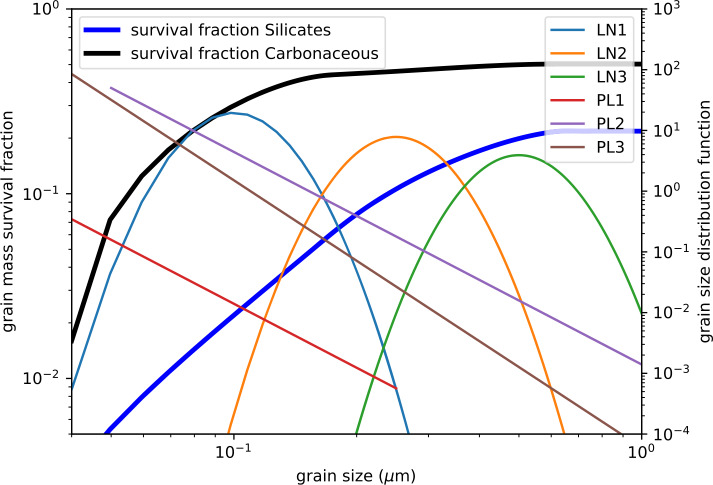
Survival rates for silicate and carbonaceous grains (thick lines and left axis) as a function of grain size (radius). LN and PL are log-normal and power-law grain size distributions (thin lines and right axis) with different parameters considered by Slavin et al. (2020) in the calculation of the overall survival rates for silicates and carbonaceous. Reproduced with permission from Slavin et al. (2020), copyright by AAS

There are additional possible phenomena that may lead to different ISD grain compositions, namely if ISDs carry an imprint of their source locations and are not catastrophically destroyed by the time they reach IMAP. While grains are subjected to shock wave-enhanced sputtering, collisions, and sublimation, supernovae and asymptotic giant branch stars have been suggested to be a source of ISD grains that preserve their formation composition (Slavin et al. 2020). Through modeling the formation and dynamics of dust grains, it was found that supernovae may efficiently produce ISD (Slavin et al. 2020) and for initial size distributions which include submicron-sized grains, approximately 10%–20% of silicate grains and 30–50% of carbonaceous grains may survive catastrophic destruction. Figure 5 shows the expected survival rates of grains from supernovae as a function of grain size, which IMAP directly tests with constraints on the size and compositional distribution of ISD. The Cassini ISD observations, on the other hand, are not consistent with pristine circumstellar dust, exhibiting a more homogeneous composition that “can be explained by destruction, recondensation, and equilibration processes in the ISM” (Altobelli et al. 2016).

IMAP can test these ISD evolution characteristics by directly measuring grain elemental and isotopic abundances. The ISD mass distribution previously measured by Ulysses was surprising because (a) it has far more large particles (radii ≳1 $\mu $m than indicated by stellar extinction measurements, and (b) the mass of heavy elements required far exceeds that available based on the local density of the interstellar gas (Draine 2009). Note, a subsequent analysis suggests a subset of the largest particles identified as ISD by Ulysses may not be of an interstellar origin (Baalmann et al. 2025). Addressing this question also has overlapping scientific and measurement goals with those described in Sect. 2.1 which focuses on the elemental and isotopic composition of the ISM. Table 3 summarizes the relevant scientific questions discussion in this subsection.

**Table 3:** Relevant science questions, IMAP measurements, and model inputs for Sect. 2.3

Relevant questions	How is the ISM elemental abundance partitioned into gas and dust? Are silicate and carbonaceous material mixed within each ISD grain, or are there two distant grain populations each dominated by a single compositional type? What role does the plasma play in abundance ratios? Are there signatures of supernova remnants?
IMAP Measurements	IDEX: Mass distribution of ISD. Elemental and isotopic ratios to identify compositional grain families. IMAP-Lo: Abundances of H, He, O, Ne. CODICE: ^22^Ne/^20^Ne ratio.
Model input	Link the observed elemental distributions to a comprehensive accounting of the total ISM elemental abundances. Hydrodynamical calculations to determine dust survival rates based on mass and composition. Investigate size-dependent effects on silicates and carbonaceous grains.

### What Is the Structure of the Heliosphere’s Local Interstellar Environment?

In addition to the compositional characteristics of the pristine LISM, here we discuss how IMAP peers into the structure of our local interstellar environment and provides insight complementary to astrophysical observations, summarized in Table 4. The interstellar environment of the heliosphere consists of hot and tenuous Local Bubble material extending ∼100-200 pc from the Sun (Lallement et al. 2003; Breitschwerdt et al. 2017; Zucker et al. 2022). However, the Sun is inside a patch of colder partially neutral interstellar cloud (Lallement et al. 2005; Slavin and Frisch 2002; Frisch et al. 2011) Hubble Space Telescope (HST) provides access to many absorption lines in the UV part of the spectrum, significantly expanding the capabilities of studying the structure of the LISM (Linsky et al. 1993, 2000). The lack of the Local Interstellar Cloud (LIC) absorption in a portion of the sky indicates that the Sun must be within 0.19 pc of the LIC edge (Wood et al. 2000).

**Table 4:** Relevant science questions, IMAP measurements, and model inputs for Sect. 2.4

Relevant questions	Are there differences in flow vectors and temperatures of different ISN species H, He, O, and Ne? What do they tell us about the properties of the possibly mixed interstellar cloud medium ahead of the heliosphere?
IMAP Measurements	IDEX: Speed distribution of interstellar dust particles. IMAP-Lo: Flow vectors and temperatures of ISN species H, He, O, and Ne.
Model input	Elemental and dust velocities at 1 au and how they depend on heliospheric conditions such as the time-varying interplanetary magnetic field, radiation pressure, heliospheric boundary conditions. Effects of multiple clouds on flow velocities as measured at 1 au. Effects of differential re-ionization losses of ISN species inside the termination shock based on IMAP measurements of the 3D structure of the solar wind.

Subsequent analysis of the high-resolution spectra from HST showed the presence of 15 interstellar clouds within 15 pc of the Sun, each with a different velocity vector (Redfield and Linsky 2002, 2004a,b, 2008). Furthermore, the obtained velocity vectors of the LIC and neighboring G clouds appeared to be different from the neutral flow vector in the heliosphere, as derived from consolidated observations of the He ISN flow through the heliosphere combining UV backscatter, PUI, and direct neutral gas flow observations (Möbius et al. 2004). On the side of the remote sensing observations, a debate ensued whether the 15 interstellar clouds are in fact a single cloud filling the entire neighborhood of the Sun, but with relative motion between parts of it (Gry and Jenkins 2014), or indeed separate clouds with occasional interstitial space (Redfield and Linsky 2015; Malamut et al. 2014). A more recent study takes this approach toward a detailed analysis of the LIC structure, at whose edge the solar system likely resides (Linsky et al. 2019).

This situation may also suggest that the ISN velocity vector relative to the Sun could vary over time in speed and/or direction (Frisch et al. 2013). The relative speed between the interstellar gas and the Sun, measured deep inside the solar system, has been constrained via analysis of approximately two solar cycles of the heliospheric hydrogen backscatter glow observed by SOHO/SWAN (Katushkina et al. 2015a; Koutroumpa et al. 2017; Bzowski et al. 2023). IMAP’s GLOWS instrument will provide new, contemporaneous measurements of this backscatter glow (Bzowski et al. 2025). That analysis also showed that the longitude of the interstellar H flow vector does not vary significantly from an average of $252.9^{\circ} \pm 1.4^{\circ}$ throughout the 20-year span. From interstellar He data taken by IBEX during more than a full solar cycle, an invariant inflow direction $(\lambda , \beta ) =(255.59^{\circ} \pm 0.23^{\circ}, 5.14^{\circ} \pm 0.08^{\circ})$ at the heliopause was determined (Swaczyna et al. 2022a).

However, detecting hypothetical variations in the velocity of the interstellar medium relative to the Sun is challenging for observation durations less than ∼30 years, i.e., three solar cycles. ISN atoms spend a substantial amount of time traveling between the unperturbed region of the LISM and a detector at 1 au from the Sun, more than 30 years for interstellar He and even longer for interstellar H (Bzowski and Kubiak 2020). There is a large spread in the transit times for these atoms, comparable to the travel times. If IMAP is active for approximately a solar cycle, then it might be possible to detect such variations if they exist by combining IBEX and IMAP observations over decades.

The combination of the ISN He flow parameters from the IBEX (Swaczyna et al. 2022a), Ulysses (Wood et al. 2015), and STEREO (Taut et al. 2018) observations confirmed that the local flow inside the heliosphere is statistically inconsistent with the flows in the main parts of the LIC and G cloud (Swaczyna et al. 2022b). However, the flow inside the heliosphere is consistent with a linear combination of the flows in these two clouds. The locations of the LIC and G clouds in space and their velocity vectors show that these clouds are destined to collide. Therefore, Swaczyna et al. (2022b) proposed that the heliosphere is located inside the Mixing Interstellar Cloud Medium (MICM) formed in the collision of these two clouds. Collisions between clouds may occur due to their relative motion (Vannier et al. 2025; Zucker et al. 2025). Additionally, Ulysses ISD observations indicated an anomalous period during which the interstellar flow deviated by over 50^∘^, a mystery that has yet to be resolved (Strub et al. 2015).

**Fig. 6:**
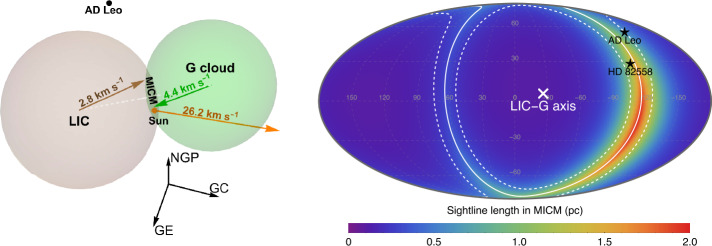
An illustrative model of the interacting LIC and G clouds (left panel) with the predicted depth of the MICM as a function of the direction in the sky (right panel). The AD Leonis and HD 82558 stars are located behind significant depths of the MICM. Reproduced with permission from Swaczyna et al. (2022b), copyright by the author(s)

The MICM’s existence is supported by a high ISN H density near the heliosphere ∼0.195 cm^-3^ (Swaczyna et al. 2020), which is approximately two times higher than the typical density (∼0.1 cm^-3^) in the LIC (Linsky et al. 2022). Furthermore, the two sightlines indicating the highest column density of ISN hydrogen are toward stars AD Leonis and HD 82558, which indicate average ISN hydrogen density along these sightlines of 0.19 cm^-3^ and 0.20 cm^-3^. The MICM explains these observations because these two stars are located within the direction where the MICM extends the most. Figure 6 presents an illustrative model of the LIC-G cloud interaction. The clouds are approximated as spheres, and thus, the interaction region has a lenticular shape extending in the direction perpendicular to the axis connecting the centers of the LIC and G clouds. Finally, the asymmetry of the ISN He distribution function along an axis consistent with the difference in the velocity flows of the two clouds (Wood et al. 2019) is explained if the ISN He near the Sun is not fully thermalized mixture of ISN He from these two clouds.

The contrast between the lack of equilibrium in the mixed region ahead of the heliosphere and different collisional coupling lengths between the plasma and ISN species may result in somewhat different flow vectors/temperatures between ISN H, He, O, and Ne. Discovering these so-far hypothetical differences requires independent determination of the flow vectors of the primary populations of these species with sufficient precision. On IBEX, this was not possible for fundamental reasons: a short interval during the year, when the ISN species are observed, results in a high correlation between the flow parameters of ISN species, obtained from observations (Schwadron et al. 2022). This results in relatively large uncertainties of the parameters, which cannot be reduced by enhancing the measurement statistics. Observations of ISN over different times during the year, enabled by the ability to change the pivot angle of IMAP-Lo, removes the parameter correlation and thus greatly reduces the large uncertainty resulting from the parameter correlation (Bzowski et al. 2022).

IMAP investigates the ISN flow dynamics with three of its instruments: the ISN flow directly with IMAP-Lo and through PUIs with CoDICE and SWAPI. IMAP-Lo observations allow for a more detailed analysis of the distribution function of ISN populations due to its pivot platform (Schwadron et al. 2025; Kubiak et al. 2023). This will allow us to study the departure of the distribution function from the Maxwell distribution. Due to the long thermalization length of ISN He in the LISM, we expect that it may not be fully thermalized in and near the MICM. Additionally, we search for a departure from the equilibrium by comparing the flow vectors of different species, reflecting the different thermalization scales of these species (Sect. 2.5). Furthermore, the IDEX observations provide information about the interstellar dust properties (Horányi et al. 2025), which in the past exhibited multiple distinct upstream flow directions (Strub et al. 2015).

### What Is the Upstream ISN Velocity Distribution?

In this section, we discuss the importance of understanding the velocity distribution function (VDF) of interstellar neutral gas (H, He, O, Ne, and other neutral species) interacting with the heliosphere, our current knowledge about the VDF from prior observational and theoretical studies, and open questions that new IMAP measurements will help to answer, summarized in Table 5.

**Table 5:** Relevant science questions, IMAP measurements, and model inputs for Sect. 2.5

Relevant questions	To what extent is the velocity distribution of ISNs non-Maxwellian? Is there evidence of temperature anisotropy in ISNs? Do the non-Maxwellian features of the VDF indicate the pristine state of the LISM, or are they the result of interactions between the LISM and the heliosphere?
IMAP Measurements	IMAP-Lo: flux of ISNs He, O, Ne as a function of direction throughout the heliosphere. CODICE and SWAPI: He pick-up-ions. IDEX: ISD flow speed at 1 au.
Model input	PUI transport within the focusing cone to back out the flow velocity. ISN transport to back out the pristine ISN flow based on the focusing and transport to 1 au, including a model of charge exchange and collisional interaction between charged and neutral species in the outer heliosheath.

The properties of interstellar neutral gas flowing from the LISM into the heliosphere play a major role in the overall interaction between the LISM and the heliosphere. The velocity vector of the LISM relative to the Sun is a central quantity that regulates this interaction. The velocity vector and the LISM temperature have been the objective of studies spanning UV backscatter observations of H (Bertaux and Blamont 1971; Thomas and Krassa 1971; Adams and Frisch 1977) and He (Weller and Meier 1974; Ajello 1978), pickup ion detection (Möbius et al. 1985; Gloeckler et al. 1992; Gloeckler 1996), and the direct measurements of ISN He (Witte et al. 1996). The IMAP-Lo instrument (Schwadron et al. 2025) will provide measurements of LISM flow parameters with improved accuracy, tracking the ISNs over $> 180^{\circ}$ in ecliptic longitude with improved angular resolution.

The most simplistic assumption one could make about the interstellar gas is that its state is close to thermal equilibrium. Then, the neutral and ionized components are in equilibrium with each other, the flow is spatially homogeneous, and the VDFs of both of the neutral and ionized components are described by the Maxwell-Boltzmann functions. However, there is substantial observational evidence indicating that the VDF of ISN gas is not Maxwellian. Analysis of interstellar He data from IBEX and Ulysses found that the bi-Mawellian VDF solution fits both datasets better than other VDF solutions (Wood et al. 2019). A preferred fit with the temperature anisotropy $T_{||}/T_{\perp} = 0.62 \pm 0.11$ was consistent with the data, and it was found that the detected VDF asymmetries are aligned with the VLISM motion relative to the Sun, suggesting it can be introduced in the outer heliosphere due to filtration processes.

Additionally, analysis of absorption lines in the spectra of nearby stars showed that in addition to thermal motion of the atoms, there must be another, turbulent, component, with a speed of at least 2 km s^−1^ (Redfield and Linsky 2004a). The spatial scale of this turbulent motion is unknown, but analysis of the behavior of the magnetic field time series from Voyager (Xu and Li 2022) suggested that there is a damping of turbulence in interstellar material below a spatial scale of $\sim 260$ au. This would leave only interstellar turbulent motions at a spatial scale much larger than this, which is comparable to the size of the heliosphere. This in turn suggests the presence of differences in the parameters of the VDF of the interstellar medium surrounding the heliosphere.

The analysis of the absorption lines in the spectra of nearby stars suggests that either the Sun is traversing a complex of relatively small interstellar clouds with the size of several parsecs each (Linsky and Redfield 2014), or is in a large cloud of warm, partially ionized material with multi-scale internal motions and gradients in the temperature, density, and ionization degree (Gry and Jenkins 2014). As previously discussed, the Sun may be traversing a region of collision between two of the nearby interstellar clouds suggested by Lallement et al. (1995), namely the LIC and G clouds (Swaczyna et al. 2022b). The velocity vector of interstellar He measured by IBEX is between the velocity vectors of these two clouds (Swaczyna et al. 2022b), and furthermore, the direction of the flattening of the temperature tensor, discovered in the IBEX-Lo and Ulysses direct sampling observations by Wood et al. (2019), approximately agrees with the relative velocity between these two clouds.

The VDF of interstellar H is expected to be non-Maxwellian. Within a kinetic modeling framework, the initially assumed Maxwellian distribution of ISN H in the LISM was found to be significantly disturbed by the charge-exchange interactions throughout the interaction region (Izmodenov et al. 2001). Notably, even at large distances from the Sun, extending beyond the bow wave (∼ 300-400 au), the VDF of ISN H deviates from Maxwellian due to hydrogen from the outer heliosphere. Non-Maxwellian distributions in the LISM were also found when comparing distributions of interstellar hydrogen between two modeling approaches, kinetic and multi-fluid (Heerikhuisen et al. 2006), along with a neutral solar wind population (Florinski and Heerikhuisen 2017). This neutral solar wind is from a charge-exchange process in the supersonic solar wind, and is highly anisotropic (parallel temperature greatly exceeding perpendicular temperature) at large distances from the Sun affecting hydrogen VDF in the LISM. IMAP, in concert with the historical IBEX data, enables us to investigate the upstream ISN velocity distribution, including the extent to which it is non-Maxwellian. Table 5 highlights the measurements and modeling inputs that would help bring scientific closure to these issues.

## How Does the VLISM Interact with the Heliosphere?

Our Sun and the solar system move through the VLISM at a relative speed of ∼25–26 km s^-1^ (see review of ISM speeds in Schwadron et al. 2022), creating an “obstacle” for the interstellar matter as the SW flows radially away from the Sun at supersonic speeds. The opposing SW and VLISM plasmas create a tangential discontinuity called the “heliopause”, where ideally the two magnetized plasmas cannot intermix, making it a challenge to measure the properties of the interstellar plasma. Furthermore, the interstellar magnetic field $\textbf{B}_{\mathrm{VLISM}}$ is observed to drape around the heliosphere, which further perturbs the dynamics of charged particles as discussed in Sect. 3.2. However, this interaction is more complex than this idealized situation due to the presence of neutral atoms in the partially ionized interstellar medium. Because they have no charge, neutral atoms can cross the heliopause and exchange information both ways, creating a unique interaction that extends from a hundred to roughly a thousand au from the Sun. Specifically, momentum and energy exchange between the heliosphere and LISM shapes this electromagnetic interaction.

Within our heliosphere, interstellar neutrals − responding to gravity, radiation pressure, and charge exchange− and dust − responding to gravity, radiation pressure, and electromagnetic forces− are filtered and processed, and thus their abundances vary throughout the heliosphere. The manner in which our heliosphere processes and filters this LISM has many open questions that we outline in this section. We highlight important facets of this interaction that IMAP is uniquely qualified to investigate. Specifically, we highlight how the LISM is modified by its interaction with the heliosphere, while the scientific questions relating to how the heliosphere responds to this interaction are primarily covered in Reisenfeld et al. (2026).

### How Does the Heliosphere Affect the VLISM?

In this section, we discuss how the presence of the heliosphere affects the VLISM through the transfer of ionized particles via neutral atoms and how dynamic SW conditions affect the nearby VLISM, summarized in Table 6. We specifically highlight how this interaction perturbs the VLISM and plays an important role in understanding the upstream conditions of the LISM via measurements at 1 au. A more extensive discussion on how this interaction affects the heliosphere is provided in Reisenfeld et al. (2026).

**Table 6:** Relevant science questions, IMAP measurements, and model inputs for Sect. 3.1

Relevant questions	How does the exchange of mass and momentum occur between the solar wind and VLISM? What material leaves the heliosphere? Where does this interaction stop? What fraction of momentum transfer occurs due to filtration of ISD?
IMAP Measurements	SWAPI/CODICE: outgoing plasma in the heliosphere. IMAP-Lo: Primary and Secondary ISN populations. IDEX: mass distribution of ISD.
Model input	Global kinetic-MHD modeling of the SW-VLISM interaction with better constrained VLISM properties.

#### Exchange of Mass and Momentum: Dust, Neutrals, and Plasma

The existence of neutral particles in the VLISM produces an exchange of mass, momentum, and energy across the heliopause, significantly changing the size, shape, and thermodynamic properties of the solar and interstellar plasmas. As interstellar neutrals enter the heliosphere, they transfer electrons to SW-ionized particles via charge exchange collisions (mostly between H and H^+^). The scattering angles in these collisions are very small, resulting in minimal change in momentum of the particles. Therefore, the SW ion is neutralized and continues its original trajectory, while the newly ionized neutral particle becomes captured by the local SW magnetic field. This process creates an ENA (from the original SW ion) and a PUI (from the original neutral particle). Due to the initial relative difference in velocities of the SW plasma and interstellar neutrals (∼400–800 km s^-1^ vs ∼25–26 km s^-1^, respectively), the PUI gains energy via the pickup in the motional electric field, producing a ring beam, shell, and eventually filled shell of PUIs (Vasyliunas and Siscoe 1976) in the supersonic SW that dominates the internal pressure of the plasma beyond 10-20 au (McComas et al. 2017).

**Fig. 7:**
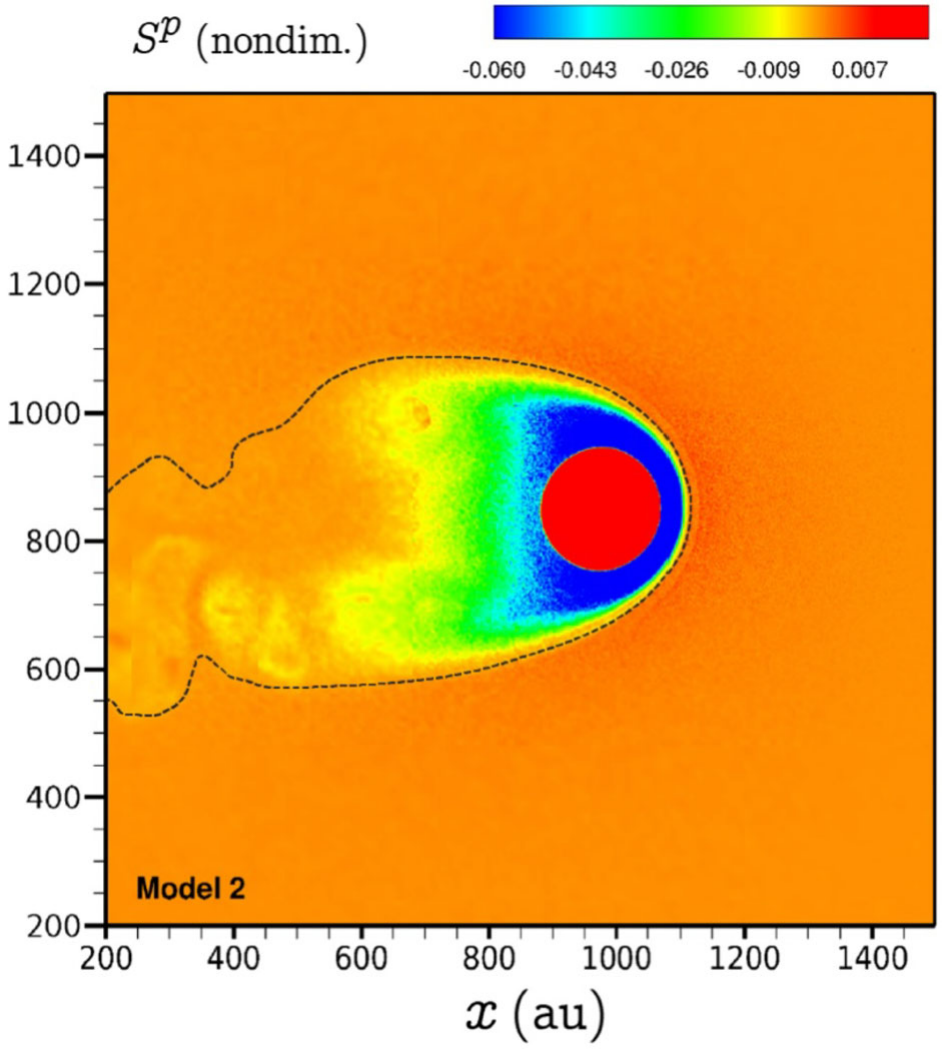
2D distribution of the plasma pressure source term, $S^{p}$, in the meridional plane from model $S2$ in Fraternale et al. (2023) including pickup ions and electrons as separate fluids, with collisional source terms. The black dashed line shows the position of the HP tracked using the level-set method. Reproduced with permission from Fraternale et al. (2023), copyright by the author(s)

The gain of momentum by newly injected PUIs leads to a loss of momentum of the SW plasma and production of ENAs. This effectively increases the plasma pressure inside the heliospheric termination shock (HTS), reduces pressure just inside the heliosheath via loss by charge exchange, and increases pressure in the VLISM, as shown in Fig. 7. Effectively, this leads to a shrinking of the HP and expansion of the HTS (Malama et al. 2006; Pogorelov et al. 2016). Moreover, the exchange of particles starting with the interstellar neutrals partially symmetrizes the heliosphere (Izmodenov et al. 2009; Pogorelov et al. 2007, 2009). Most of the ENAs in the heliosphere generated through charge exchange easily escape the heliosphere and modify the thermodynamic properties of the VLISM (see Sect. 3.1.2).

Interstellar dust is perturbed by the heliosphere, and the smallest grains are deflected around the heliopause (Czechowski and Mann 2003; Slavin et al. 2012), while larger grains can transit through the heliosphere. Observations of ISD therefore provide a unique window into our heliosphere’s interaction (e.g. Grün et al. 1994; Strub et al. 2019; Sterken et al. 2023). Figure 1 illustrates this process, also discussed in Sect. 3.3. This deflection imparts dynamic pressure on the heliosphere and is not well constrained due to limited ISD measurements, particularly in the smaller size range. Combining IDEX observations of the larger size grains with models of the full ISD size distribution and electromagnetic interaction provides an improved understanding of the ISD momentum exchange with the heliosphere.

#### What Material Leaves the Heliosphere?

SW ions can be neutralized through charge exchange collisions with ISN atoms penetrating the heliosphere. Thus, these collisions lead to the neutralization of the SW ions both in the supersonic SW and the hot heliosheath plasma, which easily escape the heliosphere due to their large mean free paths (i.e., the 1/e folding distance over which a neutral particle can travel before ionizing again). The mean free path of ENAs inside the heliosphere is hundreds of au due to the relatively low density of ions. However, once these ENAs escape the heliosphere and enter the VLISM, the plasma density increases by a factor of ∼40 across the heliopause (Gurnett et al. 2013; Pogorelov et al. 2017b; Gurnett and Kurth 2019), dramatically decreasing the ENA mean free paths to 100’s of au or lower, depending on their kinetic energy (Zirnstein et al. 2016). The “deposition” of outward propagating ionized ENAs (i.e., secondary PUIs) increases the thermal energy of the VLISM plasma, and higher energy ENAs (≥5 keV) modify the VLISM plasma upstream of the bow wave (Zank et al. 2013; Pogorelov et al. 2017a). This change in the plasma beta of the VLISM plasma upstream of the bow wave weakens the shock, creating a bow wave (McComas et al. 2012a; Zank et al. 2013; Heerikhuisen et al. 2014). This process also reduces the momentum of the VLISM plasma moving towards the nose of the heliosphere. Overall, the escape of ENAs leads to a shrinking of the heliosphere and modification of the VLISM plasma properties (by heating and increasing the plasma beta) impacting the heliosphere.

#### Where Does the Solar-Interstellar Interaction Stop?

The solar-interstellar interaction has no discrete boundary due to the continual deposit of PUIs inside the heliosphere via ionized interstellar neutrals and the resulting ENAs that escape the heliosphere at a wide range of energies; the higher the ENA energy, the farther it can travel into the VLISM and deposit energy. Global simulations of the solar-interstellar interaction, however, can offer predictions for the minimum distance into the VLISM that the interaction affects it. This requires tracking the mass, momentum, and energy source terms (Baranov and Malama 1993a; Izmodenov et al. 2003; Heerikhuisen et al. 2006, 2019; Fraternale et al. 2023). As demonstrated in these studies, the exchange of energy resulting in the modification of the VLISM plasma can reach at least ∼400 au upstream of the Sun, and far down the heliotail ahead of the bow wave. See Reisenfeld et al. (2026) for a relevant discussion on the heliotail in the context of IMAP. This length scale of 100’s au also depends on the strength and orientation of the ISMF.

**Fig. 8:**
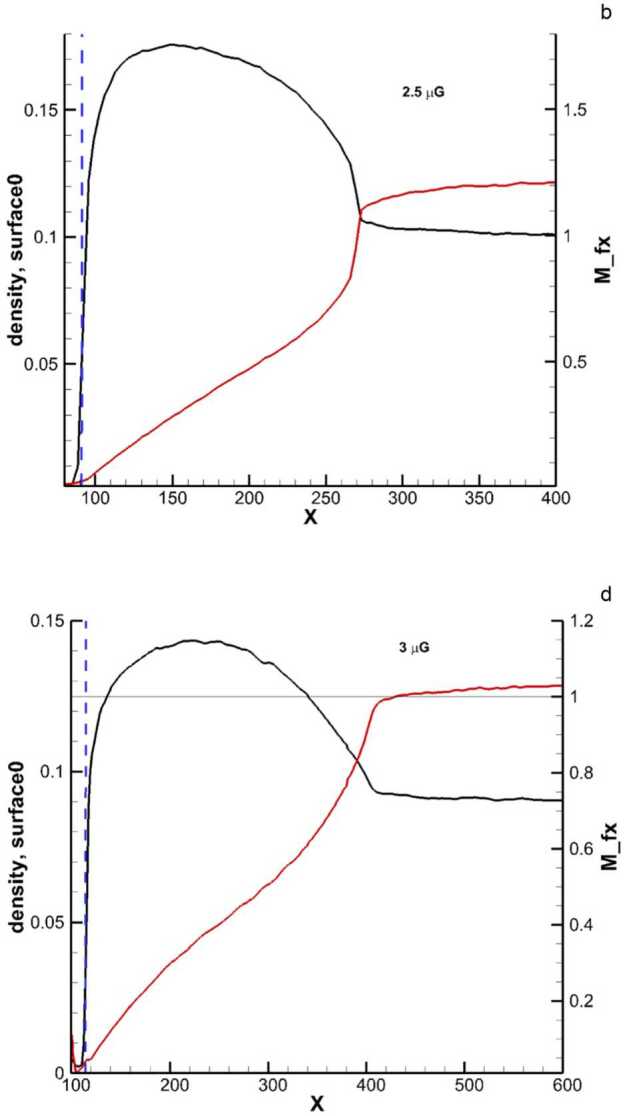
Simulated plasma density (black lines) and fast magnetosonic Mach number (red lines) along the near-upwind direction. The vertical blue dashed lines show the HP position. Note that with a field strength of 3 $\mu $G, the VLISM upstream of the bow wave is barely supersonic. Reproduced with permission from Pogorelov et al. (2017b), copyright by the author(s)

For weaker magnetic fields, the bow wave is more like a shock that is closer to the heliosphere, but for stronger ISMF the shock becomes an extended wave reaching hundreds of au further upstream into the VLISM (Heerikhuisen et al. 2014; Pogorelov et al. 2017b), as demonstrated in Fig. 8. This is also partially caused by the change in plasma and neutral densities required to maintain a consistent pressure balance on the heliopause. The LISM magnetic field estimated from the IBEX ribbon is ∼2.9 $\mu $G, and oriented towards (227^∘^, 35^∘^) in ecliptic J2000 coordinates (Zirnstein et al. 2016). This yields a bow shock/wave that begins more than 400 au upstream of the Sun, especially when the Mach number is already decreasing at >600 au from the Sun (Pogorelov et al. 2017b). However, heliospheric asymmetries observed by Voyager suggest a stronger (4 $\mu $G) and less inclined magnetic field to the flow direction (see Opher 2016), which would affect the size of the VLISM region influenced by the heliosphere This is a highly non-linear interaction, thus requiring IMAP to help us to better understand the VLISM properties which are used as LISM boundary conditions in global simulations. Comprehensive modeling in concert with IMAP observations will enable us to better constrain the extent to which the VLISM is perturbed by our heliosphere, which will in turn allow us to better constrain the pristine properties of the LISM.

#### Outgoing Plasma Leaving the Heliosphere

The SWAPI (Rankin et al. 2025) instrument onboard IMAP measures the properties of the core SW plasma (H^+^ and He$^{\mathrm{$+$$+$}}$) and interstellar He^+^ PUIs over energies ∼0.1–20 keV/q. CODICE-Lo (Livi et al. 2026) measures the core SW, PUI, and suprathermal (ST) ions ranging from ∼3-60 amu over energies ∼0.5–80 keV/q, and CODICE-Hi will measure ST ions greater than 60 amu from ∼0.03–5 MeV/nuc as well as energetic electrons from ∼20–600 keV. Finally, the SWE instrument measures the properties of the thermal electron population in the SW (Skoug et al. 2026). Thus, these complementary instruments together cover a wide energy/charge(nuc) range of particles originating from the Sun as well as interstellar PUIs generated by the ionization of interstellar neutrals propagating into the heliosphere from the VLISM. The measurements, made at L1, inform us of the particle distributions that propagate into the outer heliosphere.

Evidence of particles that exit the heliosphere can then be measured by the IMAP ENA imaging suite: IMAP-Lo (Schwadron et al. 2025), IMAP-Hi (Funsten et al. 2026), and IMAP-Ultra (Gkioulidou et al. 2026), as discussed further in Reisenfeld et al. (2026). The deposit of energetic particles in the VLISM will, after some time, depending on the energy and ionization cross section with the local VLISM plasma, form secondary ENAs. Some of them travel back into the heliosphere; this is called the secondary ENA mechanism (McComas et al. 2009a; Heerikhuisen et al. 2010). The secondary ENAs that re-enter the heliosphere and make it back to IMAP are measured over a wide range of energies from ∼0.1 to >100 keV. The complementary measurements of the outgoing SW/PUI/ST particles and incoming secondary ENAs, delayed by roughly a few years down to a few months, allow us to better understand the size and scale of the heliosphere (Reisenfeld et al. 2021; Zirnstein et al. 2022b), as well as the rate of ionization in the VLISM as a function of the densities of different VLISM plasma species.

### What Is the Pristine Interstellar Magnetic Field Configuration and How Does It Drape over the Heliosphere?

Observations of the IBEX Ribbon have demonstrated that the direction of the interstellar magnetic field draped over our heliosphere deviates from the direction in the pristine VLISM (McComas et al. 2009a). Such draping has been investigated via in-situ measurements from the Voyager mission and also via remote sensing of ENAs from the heliospheric boundary regions and of ISN crossing into the heliosphere (for a recent review see Galli et al. 2022 and references therein). The position of the ENA-Ribbon (see Reisenfeld et al. 2026 and Fig. 11) detected with IBEX led to an inferred magnitude (∼2.9 $\mu $G) and direction ($227^{ \circ}, 35^{\circ}$) of the pristine $\textbf{B}_{\mathrm{VLISM}}$ far from the Sun (Zirnstein et al. 2016; Grygorczuk et al. 2011). This has also led to the conclusion that Voyager 1 is still observing the draped $\textbf{B}_{\mathrm{VLISM}}$ (Galli et al. 2022; Rankin et al. 2023), though there remain uncertainties with how the compressed magnetic field near the heliopause unfolds towards the pristine interstellar medium.

The observation of secondary ISN also constrains the direction of $\textbf{B}_{\mathrm{VLISM}}$ near the heliopause. This local direction introduces an asymmetry in the heliosphere and deflection of the plasma flow in the vicinity of the heliopause (see Sect. 3.2.2 for more details). As a result, neutral oxygen (Baliukin et al. 2017) and helium (Swaczyna et al. 2023a) atoms that originated from charge-exchange in this region deviate in their average velocity from the pristine VLISM bulk velocity.

**Fig. 9:**
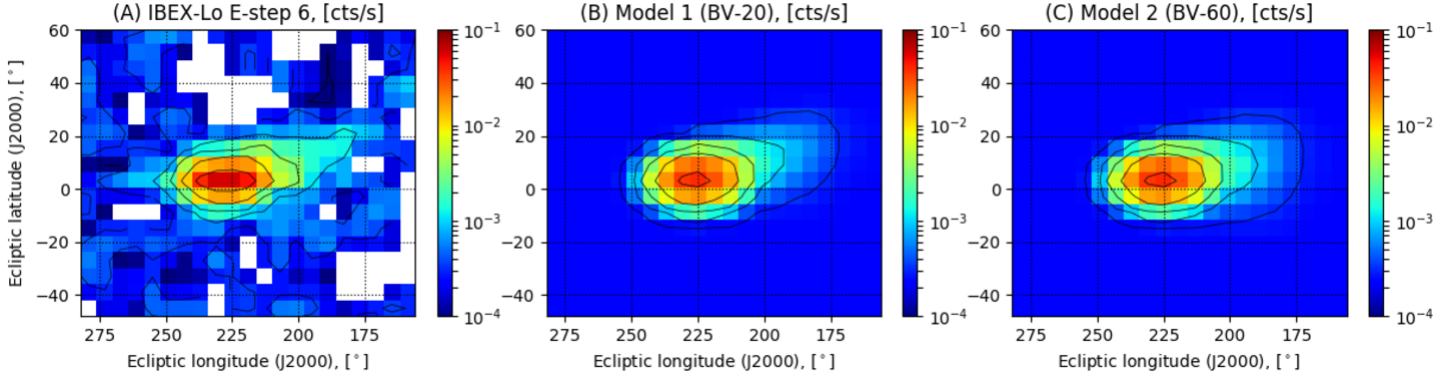
(A) Skymap (in ecliptic coordinates) of count rates due to O & Ne atoms as observed with IBEX-Lo (Park et al. 2015); (B) skymap according to model predictions by Baliukin et al. (2017) for $|\textbf{B}_{\mathrm{VLISM}}|$ = 4.4 $\mu $G and angle between $\textbf{B}_{\mathrm{VLISM}}$ and $\textbf{V}_{\mathrm{VLISM}} = 20^{\circ}$, (C) skymap according to model predictions by Baliukin et al. (2017) assuming $|\textbf{B}_{\mathrm{VLISM}}|$ = 3.75 $\mu $G and angle between $\textbf{B}_{\mathrm{VLISM}}$ and $\textbf{V}_{\mathrm{VLISM}} = 60^{\circ}$. Reproduced with permission from Galli et al. (2022), copyright by the author(s)

As demonstrated in Fig. 9, the spatial distribution of observed secondary ISN inside the heliosphere changes with parameter settings for the $\textbf{B}_{\mathrm{VLISM}}$. These changes are rather subtle (middle and right panel in Fig. 9). The better statistics and map coverage achieved with IMAP-Lo compared with IBEX-Lo will therefore prove beneficial to make use of secondary ISN as another indicator for $\textbf{B}_{\mathrm{VLISM}}$ and its draping direction.

The globally distributed flux (GDF, McComas et al. 2009a) of ENAs so far are not considered to carry signatures of interstellar magnetic field draping because these ENAs are more homogeneously distributed across the sky than the Ribbon and they have been considered to originate mostly from PUIs in the heliosheath (see Galli et al. 2022 for a review). If this interpretation of GDF origins turns out to be incomplete (Fuselier et al. 2021; Galli et al. 2023) and a part of them originates from ions neutralized outside the heliopause, this topic needs to be re-examined with the new IMAP observations. Table 7 summarizes the relevant sciences goals discussed in this subsection.

**Table 7:** Relevant science questions, IMAP measurements, and model inputs for Sect. 3.2

Relevant questions	Are there indications in the Ribbon ENAs and the GDF ENAs for a different direction of the draped B-field close to the heliopause?
IMAP Measurements	IMAP-Lo: Primary vs. secondary ISN populations of various species to determine the B-V plane. IMAP-Hi: center of ribbon fit.
Model input	Magnetic topology of the magnetic field as it drapes over the heliosphere. The dependence of ENA emissions detectable at 1 au on this draping. Modeling of the synthesis of secondary ISN populations due to collisions beyond the heliopause

#### Constraints on the Interstellar Magnetic Field Direction from the Center of the Ribbon

From the first observations of the ENA ribbon observed by IBEX, it was determined that the direction towards the ribbon peak locations is approximately towards IBEX’s look direction that is perpendicular to the VLISM magnetic field draped around the heliosphere, in agreement with global heliosphere models (Schwadron et al. 2009). It was initially determined that the center of the ribbon (i.e., the center of a nearly-circular structure super-imposed over the sky) is (longitude, latitude) = (221^∘^, 39^∘^) (Funsten et al. 2009). If the ribbon peak locations are perpendicular to the local field direction, then the center is likely closely aligned with the direction of the ISMF in the VLISM.

In the following years, a more comprehensive determination of the center of the ribbon as a function of ENA energy was performed, by fitting a circle and ellipse to the ribbon in a frame where the ribbon is approximately centered on a polar coordinate (see Fig. 2 in Funsten et al. 2013), yielded a more robust, energy-averaged center of the ribbon of (219^∘^, 40^∘^), with uncertainties on the order of 1^∘^ (Funsten et al. 2013). This was determined from ENA maps time-averaged over 2009-2011, which reduced the fluctuations in the ENA maps, and the ribbon was found to have a slightly elliptical shape.

**Fig. 10:**
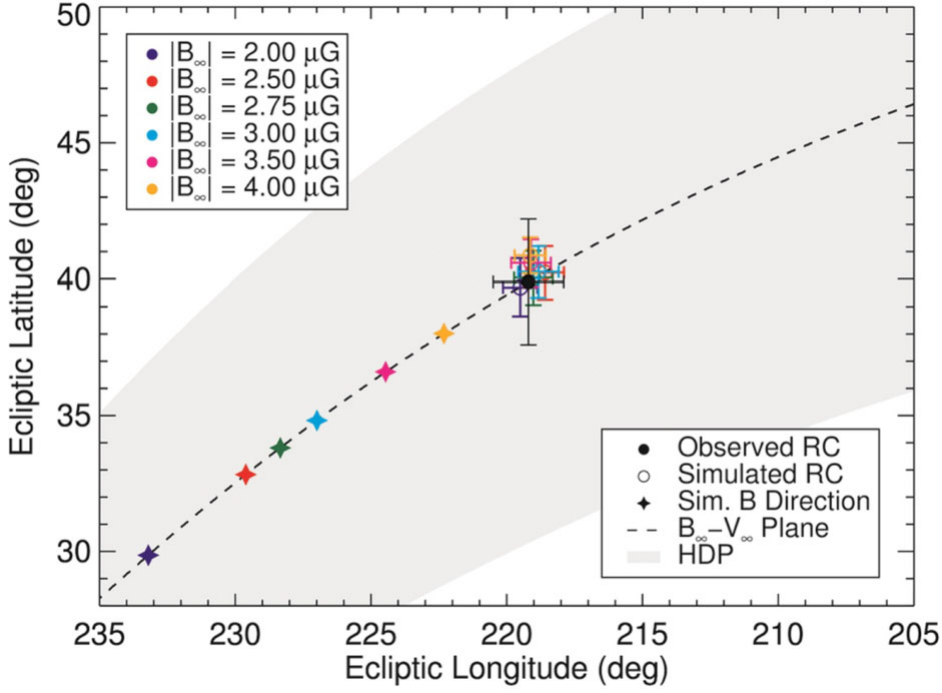
Modeled and observed ribbon centers and the corresponding ISMF. Each $|B_{\infty}|$ requires different ISMF directions (stars) to produce the same ribbon center (RC) (open circles), within the uncertainty of the IBEX measurements (black filled circle). The centers are weighted-averaged over the IBEX-Hi energy passbands. The $\mathbf{B}$-$\mathbf{V}$ plane is shown as the black dashed curve. The HDP, accounting for uncertainties, is the gray shaded region. Reproduced with permission from Zirnstein et al. (2016), copyrighty by AAS

At the time of this analysis, it was not yet apparent if the ribbon center was equivalent to the vector of the pristine ISMF, i.e., the ISMF vector unaffected by the presence of the heliosphere. The only way to determine this is through comparisons with 3D, global MHD/kinetic models of the heliosphere. Zirnstein et al. (2016) utilized a 3D global model of the heliosphere, assuming different pristine LISM magnitudes and directions, set as outer boundary conditions at 1000 au from the Sun, to narrow down the pristine field vector that produces a simulated ribbon at each IBEX-Hi energy passband that best matches the observations. From this work, it was discovered that the pristine ISMF direction lies on the same plane as the ribbon center, i.e., the $\textbf{B}$-$\textbf{V}$ plane. The weaker the pristine ISMF magnitude, the farther from the ribbon center the pristine ISMF direction lies along the $\textbf{B}$-$\textbf{V}$ plane, as seen in Fig. 10. This allowed Zirnstein et al. (2016) to find the best field direction to match IBEX ribbon center data for a variety of pristine ISMF magnitudes. Then, after performing a chi-square analysis to minimize the fit of the modeled ribbon to the IBEX ribbon, the pristine ISMF magnitude was found to be 2.9 $\mu $G with direction (227^∘^, 35^∘^). The pristine ISMF direction is approximately 8^∘^ away from the ribbon center along the $\textbf{B}$-$\textbf{V}$ plane towards the nose ($\textbf{V}$) of the heliosphere, and not the same as the ribbon center due to the draping effects of the ISMF around the heliosphere.

After nine years of IBEX ribbon observations, a follow-up comprehensive study of the temporal variability of the ribbon as a function of energy was undertaken (Dayeh et al. 2019). After performing fits to the ribbon over time and energy, they found that the ribbon centers are fairly stable over time (similar to time-dependent ribbon models from Zirnstein et al. (2023) with the same pristine ISMF vector as shown above), with only small temporal variations possibly related to SW variability. It was also found that the energy and time-averaged ribbon centers exhibit two trends: one that follows the $\textbf{B}$-$\textbf{V}$ plane (Zirnstein et al. 2016), and another that follows the heliographic meridian (Swaczyna et al. 2016), with a visible change in the trend at ∼2 keV.

IMAP-Hi, with its two camera heads, provides a significant improvement in the counting statistics, angular resolution, and temporal cadence of ENA measurements. This improves the determination of the ribbon structure, and the best functional forms to fit to the ribbon to determine its center as a function of energy and time. Moreover, the cross-over from IBEX to IMAP operations enables us to study the ribbon center (and thus the pristine ISMF vector) over two, different solar cycles. See Reisenfeld et al. (2026) for additional discussion on the interpretation of ENAs measured by IMAP in this context.

#### Constraints on ISM B-V Plane from Primary and Secondary Neutral Populations

The bulk flow velocity (V) and magnetic field (B) in the very local interstellar medium are vectors defining the B-V plane, a plane governing the distribution of the external forces shaping the heliosphere. The external boundary conditions of the heliosphere are symmetric with respect to this plane. Multiple studies (Izmodenov and Alexashov 2020; Ratkiewicz et al. 1998) showed how the relative orientation of these two vectors impacts the position of the heliopause and the shape of the heliospheric nose. The deformation of the heliopause is reflected in the interstellar plasma flow and, thus, in the flow of the secondary ISN population (McComas and Schwadron 2014).

The analysis of the Doppler shifts of the ISN hydrogen glow by the SWAN experiment on SOHO showed that the derived inflow direction of the ISN hydrogen is shifted by ∼4^∘^ from the inflow direction of the ISN helium (Lallement et al. 2005). The plane defined by ISN hydrogen and helium flow directions defines the hydrogen deflection plane (HDP). Due to the small angular distance between these directions, this plane is relatively uncertain. The SWAN observations did not allow for the separation of the primary and secondary populations. Therefore, the observed flow velocity describes a weighted mean between the flows of the two populations.

**Fig. 11:**
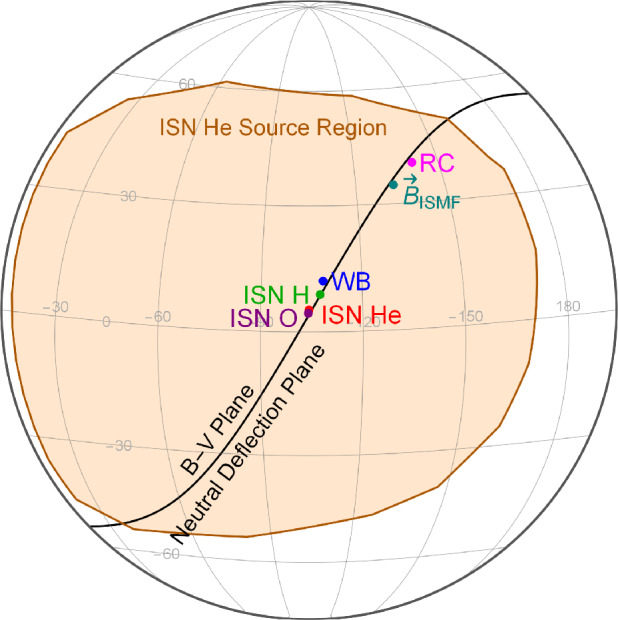
Co-planarity of the ISN flow directions: oxygen (purple), helium (red), hydrogen (green), and Warm Breeze (blue) with the ribbon center (RC) and interstellar magnetic field ($\vec{B}_{\mathrm{ISMF}}$) compared with the Warm Breeze source region (brown shaded area). Reproduced with permission from Bzowski et al. (2019), copyright by AAS

The inflow direction of the Warm Breeze discovered in the IBEX-Lo observations did not align with the B-V plane (Kubiak et al. 2014). Consequently, several hypotheses on the origin were formulated. Later analyses with more IBEX-Lo observations reduced the uncertainties of the inflow directions (Bzowski et al. 2015; Kubiak et al. 2016), and the inflow directions were found co-planar with the ISN hydrogen inflow direction, the IBEX ribbon center (Funsten et al. 2013), and the interstellar magnetic field found from the analysis of the IBEX ribbon (Zirnstein et al. 2016). Figure 11 shows the co-planarity of these directions with the region of the sky from which the secondary ISN He atoms originate as observed by IBEX.

IMAP-Lo enables a reduction of the uncertainty of the flow directions via its pivot platform (Schwadron et al. 2025), especially those connected to the “correlation tube” (Schwadron et al. 2022; Bzowski et al. 2022). Reducing these uncertainties also allows for better determination of the orientation of the B-V plane.

### How Is the ISM Filtered and Processed by the Heliosphere?

The interaction of pristine populations of ISN species with the heated and decelerated interstellar plasma around the heliosphere leads to the creation of secondary ISN populations with properties that differ from those of the pristine population. This effect has been postulated in pioneering models (Baranov and Malama 1993b), and a telltale difference between the apparent inflow parameters of ISN H and He was reported already in 1990s (Lallement and Bertaux 1990). Discovery of the secondary population of ISN He (Bzowski et al. 2012; Kubiak et al. 2014) underscores the importance of the interaction between the neutral and ionized populations beyond the heliopause.

All ISNs are expected to produce secondary populations. However, the sources of the secondary neutral atoms are different between different species. Secondary H is produced by charge exchange between pristine ISN H atoms and perturbed plasma protons (highlighted in Fig. 1). Secondary He originates from interstellar He^+^ due to charge exchange not with the abundant H population, but with ISN He, due to the large difference between the charge exchange cross sections. Hence, the secondary H is a marker of the perturbed interstellar proton population, and secondary He of the perturbed He^+^ (Bzowski et al. 2012).

Secondary O atoms are created by charge exchange between oxygen ions and hydrogen atoms$:\mathrm{O}^{+} + \mathrm{H} \rightarrow \mathrm{O}_{\mathrm{sec}} + \mathrm{H}^{+}$ in the disturbed interstellar plasma outside the heliopause. These secondary O atoms are expected to carry information about plasma protons in a region where they were created, and, therefore, they are a supplementary source of information on the physical state of the plasma in front of the heliopause. They were predicted theoretically (Izmodenov et al. 1997) and their filtration through the heliosphere boundary has been modeled by accounting for the effects of the charge-exchange and electron impact ionization processes (Izmodenov et al. 1999). IBEX-Lo measurements provided the first discovery of secondary O atoms (Park et al. 2016, 2019), which has allowed for subsequent detailed quantitative analyses of IBEX-Lo O fluxes (Baliukin et al. 2017).

Details of the production of secondary Ne remain to be studied. However, due to strong affinity between noble species it can be expected that the main source will be charge exchange between interstellar Ne^+^ and ISN He. Thus, secondary Ne may be a tracer of ionized neon in the interstellar medium and, in a broader sense, of the ionization degree of interstellar plasma.

IBEX-Lo observations of ISN O and Ne were limited to the first three years of the mission. Lowering the post-acceleration voltage in 2012 (McComas et al. 2014) led to a reduction in count rates below statistical significance. Studies of the ISN O and Ne signal for these three years (2009-2011) were mainly focused on the Ne/O ratio (Bochsler et al. 2012; Park et al. 2014) and statistical analysis of their signal (Park et al. 2015, 2016). However, a four-dimensional parameter tube for the ISN O parameters has been extracted from this dataset (Schwadron et al. 2016). In that analysis, a longitude was found that is statistically consistent with the He-derived flow longitude, albeit with large uncertainties. A similar temperature for ISN O as was also found for ISN He previously, but for higher speeds than those in the He parameter tube. In addition to low statistics, the limited observation window of IBEX-Lo further increases uncertainties in the parameter tube, particularly for Ne and O. IMAP-Lo provides considerably higher statistics and is able to observe Ne and O from different locations in the Earth’s orbit to facilitate constraining their inflow speeds and longitudes (Schwadron et al. 2025).

Retrieval of the information about the interstellar plasma from IMAP-Lo measurements of ISN fluxes requires a reliable separation of the secondary and primary atoms. There are specific times during the year and IMAP-Lo pivot angles needed to minimize either the primary or the secondary population content in the observed signal (Kubiak et al. 2023). However, this insight was provided under the assumption that the primary and secondary populations have Maxwell-Boltzmann distribution functions, which is a very simplistic assumption, as illustrated by Bzowski et al. (2017, 2019) and discussed in Sect. 2.5. The separation of the populations is challenging because the ISN flow inside the heliosphere is strongly modified by the charge-exchange gain and loss processes for neutral atoms (Bzowski et al. 2017). Elastic collisions are also important in this respect (Swaczyna et al. 2021). Only a small fraction of atoms inside the heliopause can transit through the boundary region without any collisional or charge exchange interaction. Table 8 summarizes how IMAP helps resolve these issues, and further details on transport are discussed in the following subsection.

**Table 8:** Relevant science questions, IMAP measurements, and model inputs for Sect. 3.3

Relevant questions	How does the heliosphere filter interstellar material? Once filtered and inside the heliopause, how does the remaining interstellar material interact with our heliosphere?
IMAP Measurements	IDEX: Size and velocity distribution of ISD. Compare to predictions for different model beta/size values (Slavin et al. 2012; Strub et al. 2019). IMAP-Lo: Flux of IS H, He, O, Ne (Kubiak et al. 2023). IMAP-Lo: Relation of primary to secondary population – learn about B-field in ISM. Angular scattering. IMAP-Lo: Difference in flow vectors between IS H, He, O, Ne - equilibrium condition. GLOWS: retrieval of the latitudinal structure of solar wind from helioglow observations (Bzowski et al. 2025).
Model input	Modeling transport of ISN atoms through the heliosphere and modifying their distribution function by gravitational force, radiation pressure (only for H), and ionization processes. Incorporating the heliolatitudinal and temporal variations of the ionization processes inside the heliosphere

#### Transport of ISN Atoms from the VLISM to the Inner Heliosphere

Charge exchange and elastic collisions thermalize the ionized and neutral ISM. Charge exchange is effective in the thermalization as the reactants conserve most of their momenta. Contrary, elastic collision often require multiple collisions for full thermalization due to preference of small scattering angles (Schultz et al. 2008). The length scale needed for the thermalization of neutral and ionized populations is larger than the gradients of plasma flow in and around the heliosphere.

Encountering the heliosphere, the VLISM decelerates, heats, and diverts the ionized ISM, forming a bow shock or bow wave (McComas et al. 2012b; Zank et al. 2013). The neutral ISM enters the heliosphere with less significant changes while partially decoupled from the ionized ISM (Baranov and Malama 1993b). Nevertheless, ISN populations are modified by the charge exchange and elastic collisions as they pass through the VLISM.

Charge exchange is more effective on the slow portion of the population, leaving a primary distribution that is faster and cooler (Izmodenov et al. 2001). On the other hand, elastic collisions cause partial momentum exchange between populations, slowing down the primary populations by collisions with slower plasma, while speeding up the secondary populations by collisions with faster primary populations (Swaczyna et al. 2019, 2023b; Rahmanifard et al. 2023). Inside the heliosphere, the distribution functions of ISN atoms is modified by the gravitational force from the Sun and ionization losses. In addition, radiation pressure, exerted mostly on the ISN H and D and to some extent to He atoms due to resonant absorption and re-emission of the solar EUV photons (Kowalska-Leszczynska et al. 2023) further decelerates H atoms relative to heavy species (He, O, Ne). The effects of the combined action of ionization and radiation pressure for ISN H and D result in the appearance of modification of the magnitude and direction of the flow velocity of ISN H at 1 au (Bzowski et al. 1997)

The modeling of filtration processes requires an appropriate framework. A simple approach to model the ISN flow in the heliosphere is the classical hot model providing the solution of the kinetic equation for the velocity distribution function of the interstellar hydrogen atoms assuming Maxwellian distribution at the entrance to the heliosphere (Lallement et al. 1985; Izmodenov 2006). The hot models can account for gravity, radiation pressure, and ionization inside the heliosphere However, Global heliosphere models are needed to account for filtration in the outer heliospheric boundaries (e.g., Heerikhuisen et al. 2019; Izmodenov and Alexashov 2015; Opher et al. 2015; Fraternale et al. 2021). Furthermore, considering temporal and helio-latitudinal variations of the solar parameters with the solar cycles’ evolution significantly affects ISN flow through ionization processes and radiation pressure (McComas et al. 2003; Kowalska-Leszczynska et al. 2018a,b; Sokół et al. 2013; Bzowski et al. 2002; Tarnopolski and Bzowski 2009). These modeling investigations provide key context to interpret the IMAP observations and help us better understand how the ISN is filtered, as summarized in Table 8.

## How do Interstellar Material and Interplanetary Dust Affect the Inner Heliosphere?

For our third, and last, broad science question, we investigate the effects and observability of the ISM on the innermost regions of our heliosphere. IMAP is in a unique position to measure the inner-source of PUIs with SWAPI and CODICE, while simultaneously constraining the composition of grains with IDEX that would provide a yet-determined fractional contribution of inner-source PUIs. More detail on the science of IMAP’s in-situ observations of charged particles at 1 au is provided in Cohen et al. (2026), notably relevant in how Anomalous Cosmic Rays (ACRs) are created from interstellar PUIs. The open questions discussed below make the inner source PUIs themselves a fruitful target for composition studies of their source. Table 9 summarizes the relevant questions, measurements, and modeling inputs we identify to help resolve this broad science question. We divide this question into two subsections, focusing on PUIs from interstellar neutrals and PUIs from dust.

**Table 9:** Relevant science questions, IMAP measurements, and model inputs for Sect. 4

Relevant questions	How does the composition of ISNs, ISDs, and IDPs compare to the inner source of PUIs? What is the composition and inflow rate of zodiacal grains that eventually sublimate and produce PUIs very near the Sun?
IMAP Measurements	IDEX: composition of interplanetary dust, notably Li, Na, Mg, Al, Si, K, Ca, Fe. CODICE/SWAPI: composition of inner source of PUIs from H, He, O, Ne, Ar. IMAP-Lo: ISN abundances of H, D, He, O, Ne. All relevant instruments: time-variability in PUIs and their sources.
Model input	Transport of PUIs from the inner source to 1 au. Sublimation of dust grains near the Sun vs. sputtering and their heliocentric dependence. Comparison with collisional *β*-meteoroid production.

### What Is the Relative Abundance of Interstellar PUIs Compared to the Inner Source or Any Other Solar System Object-Related Sources?

Since the discovery of interstellar PUIs (Möbius et al. 1985; Gloeckler et al. 1993), they have become a key tool for diagnosing the composition of neutral gas distributions in the solar wind and, along with that, their interaction with the solar wind (Zirnstein et al. 2022a). Early on, it became evident that PUIs contained the signature of the interstellar gas composition (Gloeckler and Geiss 2001), albeit limited to species with a high enough ionization potential so that they can survive the journey to the inner heliosphere as neutral atoms. Likewise, PUIs enabled probing the neutral gas environments of comets (Gloeckler et al. 1986; Hynds et al. 1986; Ipavich et al. 1986; Neugebauer et al. 1990) and planets (Breus et al. 1989), including sputtering off bare surfaces, such as that of the moon (Hilchenbach et al. 1992; Halekas et al. 2012).

The latter source populations may interfere with the diagnostics of the interstellar gas locally, where the solar wind sweeps the related PUIs by the observing spacecraft. For observations at the Lagrangian point L1, as onboard IMAP (McComas et al. 2025), only the occasional passage of comets close to the Sun and the encounter of solar wind flux tubes connecting to Venus (Grünwaldt et al. 1997) will be relevant. Both sources will be short-lived, and culling them from the data to concentrate on PUIs from the interstellar gas is straightforward, as their locations relative to L1 are precisely known as a function of time.

**Fig. 12:**
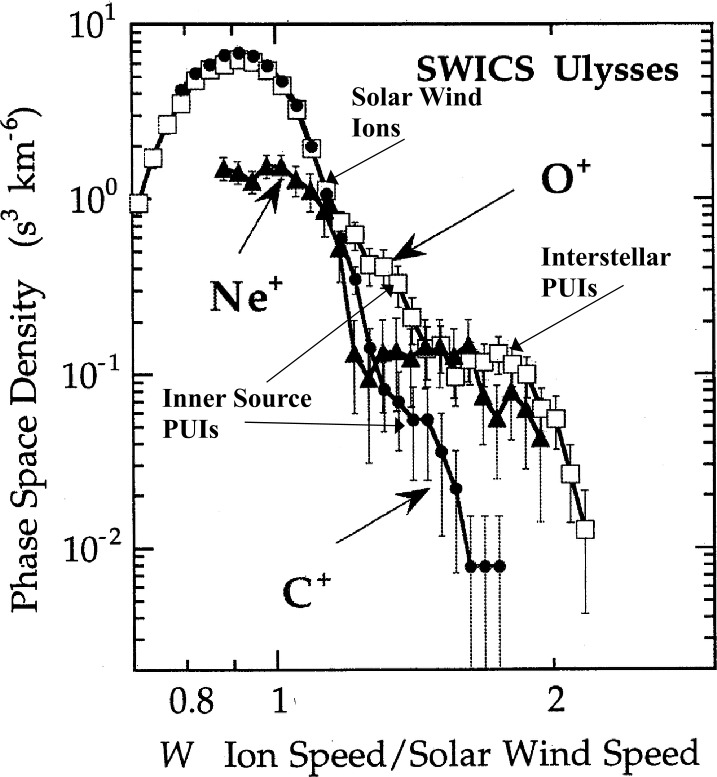
PUI velocity distributions of C^+^, O^+^, and Ne^+^, along with the respective solar wind distributions obtained with Ulysses SWICS. While Ne^+^ and O^+^ show distributions with a cut-off at 2$V_{sw}$, C^+^ is only concentrated close to the solar wind. O^+^ exhibits both components. Adapted from Gloeckler et al. (2000) with permission from JGR/Wiley

However, another extended population of PUIs was found in the solar wind, the so-called “inner-source PUIs” (Geiss et al. 1995). These PUIs manifest as a source distinctly different from interstellar neutrals due to the prevalence of low first ionization potential species, such as C, in their composition. As their hallmark, inner-source PUIs exhibit a distinctly different velocity distribution compared with interstellar PUIs, i.e., they are concentrated close to the solar wind velocity (Schwadron and Geiss 2000), whereas interstellar PUIs produce almost a flat-top distribution between the solar wind up to twice the solar wind speed (Möbius et al. 1988; Gloeckler and Geiss 1998a). In this way, the contributions of the two different sources to the observed PUI distribution of species, such as O, stemming from both sources can be separated quantitatively (Geiss et al. 1995; Gloeckler and Geiss 1998a). The velocity distributions of these two sources are shown for C^+^, O^+^, and Ne^+^ PUIs together with the respective solar wind distribution in Fig. 12. As discussed in Sect. 4.2, dust has been heavily implicated in producing inner source PUIs.

Based on the arguments above, the elemental abundances of interstellar PUIs can be assessed primarily close to the cut-off of the PUI distribution, while those of inner-source PUIs can be obtained close to the solar wind distribution. However, in some cases, C^+^ ions, which clearly belong to the inner-source PUIs, have been observed with a ring distribution near the cut-off at 1 au (Drews et al. 2016), which implies that inner-source ions may also be generated further away from the Sun at times. Therefore, it will be essential to analyze the complete PUI distributions to distinguish clearly between the different populations. Temporal variations in PUIs are another important constraint IMAP provides, as the production mechanisms of inner-source PUIs appear to vary distinctly with the solar wind conditions (Taut et al. 2015).

With its wide energy range that includes the suprathermal energies, the CoDICE instrument (Livi et al. 2026) is ideally suited to determine the abundances of heavy PUIs over the entire range of solar wind velocities. For species that contain a contribution from inner source PUIs, the transition between the two populations in the velocity distribution will be evident.

### What Is the Composition of Dust at 1 Au That Seeds the Inner Source PUIs?

As previously discussed in Sect. 2.2, the zodiacal cloud is sourced by particles shed from comets and asteroids (Nesvorný et al. 2010), dynamically evolved via gravity, radiation pressure, and Poynting-Robertson drag, and eroded by collisional grinding and sublimation near the Sun. Micron- and submicron-sized grains detectable at 1 au by IDEX (Horányi et al. 2025) within this cloud will spiral into the Sun’s atmosphere on timescales less than ∼10,000 years (Burns et al. 1979). Grains very near the Sun meet one of two likely fates (Grün et al. 1985): 1) they are collisionally fragmented, such that the majority of their fragments are so small they are ejected from the heliosphere due to the outward force of solar radiation pressure (Zook and Berg 1975) as “$\beta $-meteoroids” or b) atom-by-atom, their material is sublimated and/or sputtered away from solar radiation/wind to be subsequently ionized and become PUIs entrained in the solar wind (e.g. Mann et al. 2000). The relative amounts of zodiacal material ejected from the Sun as $\beta $-meteoroids compared to the injection of inner-source PUIs remain relatively unconstrained due to a patchwork of separate observations (Szalay et al. 2024; Pokorný et al. 2024).

There are multiple theories on how the inner-source PUIs are generated, all involving zodiacal dust. The composition of inner-source PUIs can be partially linked to the solar wind and to dust in the heliosphere (Gloeckler et al. 2000). In all models for dust-related inner source PUI production, a velocity distribution of PUIs arises that is largely concentrated close to the solar wind velocity distribution.

In one model, solar wind saturates the dust surface to be subsequently sputtered, resulting in PUIs that largely resemble solar wind composition (Schwadron et al. 2000). The presence of inner source H^+^ was not well explained by the evaporation or sputtering from grains and instead was suggested to require the absorption of solar wind ions and reemission of neutrals. However, the exceptionally high inner-source PUI flux observed contradicts the reemission process, given the observed dust density derived from zodiacal light observations (Allegrini et al. 2005) and the lack of available cross-sectional area in zodiacal grains near the Sun (Szalay et al. 2021).

In another model, solar wind ions penetrate nanometer-sized dust grains or through nanometer-scale sections of larger grains, thereby neutralizing the solar wind or generating low-charge state ions, along with some dust-related ions (Wimmer Schweingruber and Bochsler 2003). Taut et al. (2015) and Berger et al. (2015) used data from the Charge-Time-Of-Flight sensor onboard SOHO and compared the variability of inner-source and solar-wind ions, showing that inner-source PUI oxygen and carbon fluxes are directly correlated with those of the solar wind. However, this mechanism relies on a significant abundance of nanometer-sized dust grains in the very inner heliosphere. Such a population has been proposed (Czechowski and Mann 2010) and nanograins picked up and entrained in the solar wind have been suggested to be responsible for signals by STEREO’s waves instrument (Meyer-Vernet et al. 2009; Poppe and Lee 2020). However, a stable, high-residence-time and therefore high-density population of nanograins trapped near the Sun at abundances adequate to account this formation hypothesis has never been observed in-situ by a dedicated dust instrument.

Finally, direct sputtering of zodiacal grains can inject inner source ions with a composition similar to the parent grains (Quinn et al. 2018). Grain destruction during collisions close to the Sun has also been suggested to produce substantial seed ions for inner source PUIs (Mann and Czechowski 2005). “Chondritic porous” grains have been suggested to provide a seed of inner-source PUIs with compositions consistent with previous observations (Quinn et al. 2019), though a statistical in-situ characterization of micron-sized dust grain composition has not been possible before IMAP.

As discussed above, zodiacal dust is heavily implicated in the origins of inner source PUIs. The zodiacal cloud has been found to exhibit significant variability in total dust abundance within 10’s of solar radii (Szalay et al. 2025). Building on these observations, the production of inner-source PUIs has been hypothesized to exhibit variability on timescales of days to decades (Szalay et al. 2026). Constraints of inner source PUI temporal variability could therefore serve to diagnose the role of dust in inner-source PUI generation. While IMAP will not directly characterize the very near-Sun dust environment within 10’s of solar radii from the Sun, it can directly constrain the abundances and time-variability of inner-source PUIs from a stable orbit at 1 au and simultaneously constrain the composition of grains that would spiral into the Sun to produce a zodiacal inner source of PUIs, providing a critical new window into the inner source PUI formation.

## Conclusion

In this study, we have outlined scientific questions that IMAP will be uniquely suited to address relating to the properties of the pristine interstellar medium and how it interacts with our heliosphere. Specifically, we divided this interaction into the following structure and provided specific measurements and modeling inputs that would be needed to reach closure on these questions.

∘ What is the state of the pristine upstream ISM and how does it relate to its origins and evolution?
  What is the elemental and isotopic composition of the ISM?
  How does the ISM compare with interstellar and interplanetary dust?
  Is ISD destroyed and created throughout the ISM or does it carry the imprint of its origins?
  What is the structure of the heliosphere’s local interstellar environment?
  What is the upstream ISN velocity distribution?
∘ How does the VLISM interact with the heliosphere?
  How does the heliosphere affect the VLISM?
  What is the pristine interstellar magnetic field configuration, and how does it drape over the heliosphere?
  How is the ISM filtered and processed by the heliosphere?
∘ How do interstellar material and interplanetary dust affect the inner heliosphere?
  What is the relative abundance of interstellar PUIs compared to the inner source or any other solar system object-related sources?
  What is the composition of dust at 1 au that seeds the inner source PUIs?

This article provides a template for studies using IMAP data, notably condensed into the various tables throughout the manuscript, to quantitatively constrain the interaction of our heliosphere with the interstellar medium, given the unique and novel instrumentation IMAP is equipped with.

## Footnotes

1 The pristine LISM refers to the interstellar environment surrounding the heliosphere that remains unaffected by the heliospheric interaction.
